# Association between ambient temperature and injuries: A time series analysis using emergency ambulance dispatches in Shanghai

**DOI:** 10.1016/j.pmedr.2025.103177

**Published:** 2025-07-18

**Authors:** Yan Hu, Zhifeng Zhang, Li Peng, Wenjie Lu, Haotian Jiang, Jiayue Zhu, Xu Liu

**Affiliations:** a School of Health Science and Engineering, University of Shanghai for Science and Technology, Shanghai 200093, China; b Department of Health Service, Faculty of Health Service, Naval Medical University, Shanghai 200433, China; c Shanghai Medical Emergency Center, Shanghai 200233, China; d Shanghai Key Laboratory of Meteorology and Health, Shanghai Meteorological Service, Shanghai 200030, China

**Keywords:** Ambient temperature, Extreme temperature, Injury, Traffic accident, Distributed lagged nonlinear model (DLNM)

## Abstract

**Objective:**

Injuries remain a major cause of death globally amid rising health threats from climate change and extreme weather. This study examined the association between ambient temperatures and different injury mechanisms to identify vulnerable populations in Shanghai.

**Methods:**

Injury-related emergency ambulance dispatch records and corresponding meteorological data for the period 2016–2021 were obtained from the Shanghai Emergency Dispatch Center and the Shanghai Meteorological Service. A distributed lag nonlinear model (DLNM) with a quasi-Poisson distribution was applied to evaluate the association between ambient temperatures and injury-related emergency ambulance dispatches. Subgroup analyses were further conducted by gender, age group, and injury mechanisms to identify vulnerable populations.

**Results:**

Extreme temperatures were associated with increases in total injury-related emergency ambulance dispatches, as well as traffic accidents, falls, and assault injuries. Low temperatures were linked to an elevated risk of fall injuries, particularly among women aged 46 years and above. In contrast, extreme heat was associated with increased risks of traffic accidents and assault injuries among individuals aged 18–45, with assault injuries showing a particularly pronounced association among men.

**Conclusions:**

Our findings can guide prehospital emergency service departments in developing targeted interventions to reduce injury incidence and mortality during extreme temperature events.

## Highlights

• High temperatures were linked to more traffic accidents and assaults.
• More fall injuries were observed among elderly women during low temperatures.
• Young men showed more assault cases under extreme heat conditions.

## 1 Introduction

Climate change is one of the most significant health threats of the 21st century, affecting human health through multiple direct and indirect pathways (Jones, 2022; Zhao et al., 2022). The 2024 Lancet Countdown report indicates that 2023 marked the hottest year on record, with persistent droughts, lethal heatwaves, catastrophic wildfires, and extreme weather events exacerbating global health disparities and socioeconomic vulnerabilities (Romanello et al., 2024). Investigating the association between extreme temperatures and health outcomes is essential for developing evidence-based strategies to strengthen prehospital emergency response systems.

Injuries pose a major global public health burden, as evidenced by World Health Organization data indicating that annual injury-related deaths amount to 4.4 million, which accounts for 8 % of global mortality (World Health Organization, 2021). In China, injury mortality reached 46.90 per 100,000 individuals in 2021, accounting for 6.61 % of total deaths (Duan et al., 2023). Previous epidemiological studies have identified demographic (Cannada and Jones, 2006) and socioeconomic factors (Madsen et al., 2022) as contributors to injury risk. However, environmental factors, particularly extreme temperatures, remain underexplored. Increasing evidence suggests that thermal extremes are associated with elevated injury risks via direct physiological impacts and indirect behavioral changes (Dewi et al., 2024; Tian et al., 2024). A case-crossover study conducted in New York City revealed positive associations between high ambient temperatures and both unintentional and intentional pediatric injuries (Girma et al., 2023). In Vietnam, a time-series analysis further demonstrated that emergency visits related to injuries are associated with temperature, with variations observed across age and gender groups (Le et al., 2024). A study utilizing electronic hospitalization records from the SuValue database also indicated a significant association between heat and injury hospitalization (Zhou et al., 2024). Moreover, the associations between temperature extremes and injury risks differ across various injury mechanisms. For instance, a study in South Korea demonstrated a 21 % rise in traffic accident injuries during subfreezing winters (Lee et al., 2014), while New York City observed a 1.58 % increase in vehicular crashes per 1 °C temperature rise above 26.1 °C (Hou et al., 2022). Liang et al. (2021) reported elevated fall risks among elderly populations under heat stress and delayed cold effects in Ma'anshan, China. Several studies (Heo et al., 2024; Stevens et al., 2024) have reported a positive association between high ambient temperatures and increased violent behavior. Despite expanding literature, most existing studies remain focused on isolated injury analyses (Li et al., 2023; Lin et al., 2025), leaving systemic associations between extreme temperatures and prehospital emergency dispatches underexplored.

Therefore, this study aimed to conduct a comprehensive analysis of the association between ambient temperatures and injury-related emergency ambulance dispatches in Shanghai, with subgroup analyses by gender, age group, and injury mechanisms to identify vulnerable populations.

## 2 Materials and methods

### 2.1 Study area

Shanghai (31°14′N, 121°29′E) is located along China's eastern coastline. The city is characterized by a humid subtropical climate with distinct seasonal fluctuations. Summer temperatures in Shanghai peak between June and August; the monsoon season, spanning late May to early September, is characterized by persistent high humidity. The investigation of this study was carried out in the central urban area of Shanghai, including Yangpu District, Huangpu District, Xuhui District, Changning District, Jingan District, Putuo District, and Hongkou District (Fig. S1).

### 2.2 Data collection

#### 2.2.1 Injury-related emergency ambulance dispatch

Injury-related emergency ambulance dispatch data from 2016 to 2021 for the central urban area of Shanghai was obtained from the Shanghai Medical Emergency Center. The dataset encompassed information on emergency dispatches from the central urban area of Shanghai, including dispatch time, gender, age, primary complaint, medical history, disease classification and preliminary diagnosis. Injuries were identified by integrating the presenting illness history and primary complaint, in accordance with the International Classification of Diseases, 10th Revision (ICD-10) codes for injury, poisoning, and certain other consequences of external causes (S00-T98), as well as external causes of morbidity and mortality (V01-Y98), yielding a total of 239,130 cases. Records with missing information on age or gender were excluded from further analysis. Specifically, 4984 records, accounting for 2.08 % of the initial dataset, were removed, yielding a final sample of 234,146 cases.

#### 2.2.2 Environmental data

The meteorological data used in this study were obtained from the Shanghai Meteorological Service, including daily mean temperature (°C), pressure (hPa), wind speed (m/s), relative humidity (%). Air pollutant data were obtained from the Yangtze River Delta Center for Environmental Meteorology Prediction and Warning, including particulate matter with an aerodynamic diameter of less than 2.5 μm (PM_2.5_: μg/m^3^) and 10 μm (PM_10_: μg/m^3^), carbon monoxide (CO: mg/m^3^), nitrogen dioxide (NO_2_: μg/m^3^), sulfur dioxide (SO_2_: μg/m^3^) and ozone (O_3_: μg/m^3^).

### 2.3 Data analysis

The distributed lag nonlinear model (DLNM) was employed to estimate the exposure-response and exposure-lag associations between extreme temperatures and injury-related emergency ambulance dispatches (Gasparrini et al., 2010; Zhang et al., 2024). The quasi-Poisson Akaike information criteria (Q-AIC) was used to determine the optimal degrees of freedom for the model. The model was formulated as follows:

$$\log\left[E\left(Y_{t}\right)\right]=\alpha +\beta {\mathrm{Temp}}_{t}+\mathrm{ns}\left({\mathrm{Mete}}_{t},3\right)+\mathrm{ns}\left({\mathrm{Pollut}}_{t},3\right)+\mathrm{ns}\left(\mathrm{Time},8*6\right)+{\mathrm{\eta Dow}}_{t}+{\mathrm{\nu Holiday}}_{t}.$$

where $E\left(Y_{t}\right)$ is the expected number of injury-related emergency ambulance dispatches on day t; α is the intercept; β is the vector of coefficients for ${\mathrm{Temp}}_{t}$; ${\mathrm{Temp}}_{t}$ is a cross-base for temperature exposure and lag time, using a natural cubic spline with four degrees of freedom to represent temperature and three degrees of freedom to represent lag to capture nonlinear temperature and lag effects, respectively; Mete are meteorological factors, including relative humidity and wind speed, controlled by a natural cubic spline with three degrees of freedom; Pollut are pollution factors, including PM_10_, CO, and SO_2_, also controlled by a natural cubic spline with three degrees of freedom; Time represents the long-term temporal trend and the seasonal trend, controlled by a natural cubic spline with eight degrees of freedom; η and *ν* are the regression coefficients; Dow indicates the day of the week; Holiday indicates whether it is a holiday.

Once the model was established, the exposure-response relationships between ambient temperature and different mechanisms of injury-related dispatches were plotted. Further analysis was conducted through stratification by gender, age and different mechanisms of injury groups. The results were reported as relative risks (RRs) with 95 % confidence intervals (CIs). To evaluate the sensitivity of the model, we varied the degrees of freedom for time, wind speed, relative humidity, PM_10_, CO, and SO_2_, conducting a comparative analysis of their associations with injury-related emergency dispatches (Table S1). All data analyses were performed using R software (Version 4.3.1) and the “dlnm”, “mgcv” and “splines” packages.

The study was approved by the Committee on Ethics of Medicine, Naval Medical University, PLA (Approval Date: January 20, 2024).

## 3 Results

### 3.1 Descriptive analysis

**Fig. 1:**
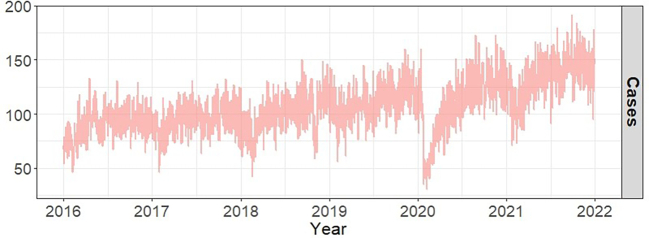
Time series distribution of total injury-related emergency ambulance dispatches in Shanghai, 2016–2021.

Table 1 presents the distribution of emergency ambulance dispatches for different injury mechanisms and environmental variables in Shanghai from 2016 to 2021. This study covered a total of 2192 days in Shanghai, during which 234,146 valid injury-related emergency ambulance dispatch records were selected. The average daily number of injury-related dispatches was 106.8 ± 23.9. The male-to-female ratio of injury-related dispatches was approximately 1:1.1, with 79 % of cases occurring in individuals aged 18–45 years and those ≥60 years. The primary injury mechanisms were falls (45.7 %) and traffic accidents (34.5 %), with an average of 36.9 ± 12.0 and 48.8 ± 11.4 dispatches per day, respectively. Fig. 1 illustrates the time series distribution of total injury-related emergency ambulance dispatches in Shanghai from 2016 to 2021. Among the environmental factors, the daily mean temperature, relative humidity, wind speed, PM_10_, SO_2_ and CO were 18.3 (°C), 71.5 (%), 0.5 (m/s), 48.9 (μg/m^3^), 8.7 (μg/m^3^) and 0.7 (mg/m^3^), respectively (Table 1). Spearman correlation analyses were conducted to assess the associations between injury-related emergency ambulance dispatches and environmental factors, with the results presented in Table S2. The environmental factor variables used in the model were significantly correlated with injury-related emergency ambulance dispatches (*p* < 0.01).

**Table 1:** Descriptive analysis of injury-related emergency ambulance dispatches and environment factors in Shanghai, 2016–2021.

	Subgroups	Percent%⁎	Mean ± Standard deviation	Min	P25	P50	P75	Max
All			106.8 ± 23.9	30	90	105	122	192
Mechanism of Injuries								
	Fall	45.7	48.8 ± 11.4	16	40	48	56	96
	Traffic accident	34.5	36.9 ± 12.0	2	29	36	44	88
	Assault	5.2	5.5 ± 2.8	0	4	5	7	17
	Else	14.6	15.6 ± 5.9	2	12	15	19	43
Environment factors								
	Mean temperature (°C)		18.3 ± 8.4	−5.2	11.1	18.9	25.3	35
	Relative humidity (%)		71.5 ± 14.2	27	62	72	82	100
	Wind speed (m/s)		0.5 ± 0.3	0	0.3	0.4	0.6	2.4
	PM_10_ (μg/m^3^)		48.4 ± 27.6	7	30	41	61	311
	SO_2_ (μg/m^3^)		8.7 ± 4.4	3	6	7	10	48
	CO (mg/m^3^)		0.7 ± 0.2	0.3	0.5	0.6	0.8	1.9

⁎ Percent% represents the percentage of each injury mechanism among the total number of injury-related emergency ambulance dispatches. PM_10_: Particulate matter with an aerodynamic diameter of less than 10 μm; SO_2_: Sulfur dioxide; CO: Carbon monoxide; Min: Minimum; Max: Maximum; P25: the 25th percentile; P50: the 50th percentile; P75: the 75th percentile.

### 3.2 Relationship between ambient temperatures and injury-related emergency ambulance dispatches

**Fig. 2:**
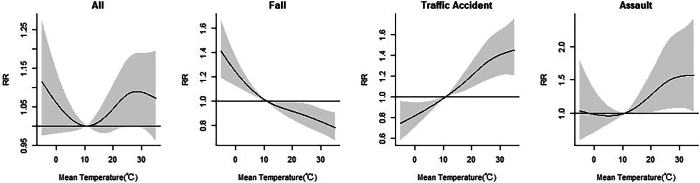
Exposure-response relationship between ambient temperature (reference = 10.8 °C) and injury-related emergency ambulance dispatches in Shanghai, 2016–2021. Shaded gray area is the 95 % CI. RR: relative risk; CI: confidence interval.

Fig. 2 demonstrates a nonlinear exposure-response relationship between ambient temperature and injury-related emergency ambulance dispatches. The temperature associated with the lowest risk of total injury dispatches was 10.8 °C, which was used as the reference. The cumulative exposure-response curve for total injuries shows an approximately U-shaped pattern. With increasing daily mean temperature, the risk of total injury dispatches decreased gradually and then increased. For fall injuries, the RRs gradually decreased as temperatures increased, with the highest RR observed at −5.2 °C (RR = 1.41, 95 % CI = 1.19, 1.67). In contrast, the RRs for traffic accidents increased progressively with rising temperatures, peaking at 35 °C (RR = 1.45, 95 % CI = 1.20, 1.75). Similarly, the RR for assault injuries also reached its maximum at 35 °C (RR = 1.57, 95 % CI = 1.02, 2.41).

### 3.3 Subgroup analysis

**Fig. 3:**
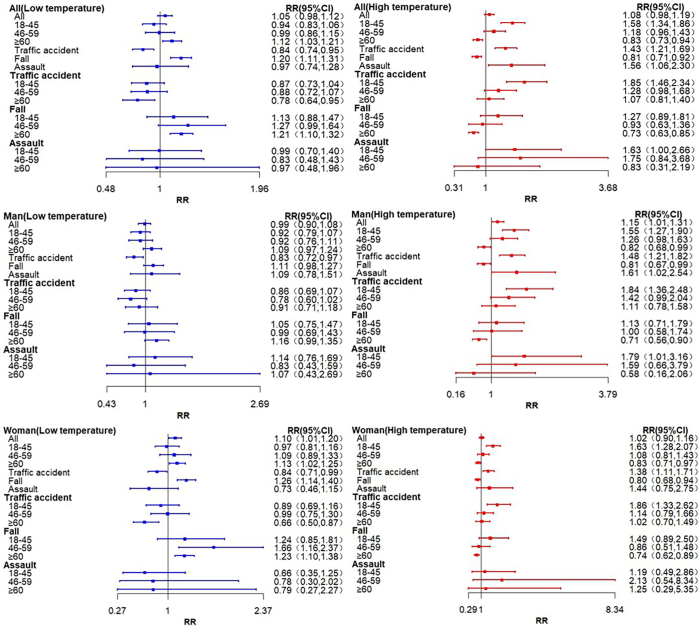
Subgroup analyses of the lagged associations between extreme temperatures (1.2 °C: the first percentile; 32.6 °C: the 99th percentile) and injury-related emergency ambulance dispatches in Shanghai, 2016–2021. RR: relative risk; CI: confidence interval.

Fig. 3 depicts the lagged associations between extreme temperatures (1.2 °C: the first percentile; 32.6 °C: the 99th percentile) and injury-related emergency ambulance dispatches across subgroups in Shanghai. Extreme low temperature was significantly associated with increased injury dispatches among adults aged ≥60 years (RR = 1.12, 95 % CI = 1.03, 1.21) and women of all ages (RR = 1.10, 95 % CI = 1.01, 1.20). Mechanistically, extreme low temperature demonstrated a robust association with fall injuries (RR = 1.20, 95 % CI = 1.11, 1.31), particularly among women aged 46–59 years (RR = 1.66, 95 % CI = 1.16, 2.37). Extreme high temperature exhibited an association with injury dispatches in the 18–45 age group (RR = 1.58, 95 % CI = 1.34, 1.86). Gender-stratified analyses revealed significant associations between extreme heat and traffic accident injuries in both men (RR = 1.84, 95 % CI = 1.36, 2.48) and women (RR = 1.86, 95 % CI = 1.33, 2.62) aged 18–45 years. Furthermore, extreme high temperatures were associated with elevated assault injuries in men aged 18–45 years (RR = 1.79, 95 % CI = 1.01, 3.16).

## 4 Discussion

This study examined the association between ambient temperatures and injury risk, with a specific focus on injury mechanisms in Shanghai from 2016 to 2021. A total of 234,146 valid injury-related emergency ambulance dispatch records were collected, with falls and traffic accidents identified as the leading causes. Previous studies have demonstrated a significant relationship between injury risk and ambient temperature, with the association curves varying substantially across different injury mechanisms. Our findings extend existing evidence by providing detailed analyses of subgroup populations across different injury mechanisms.

A significant elevation in traffic accident injury risks was observed under extreme heat conditions in our study. Similarly, Zou et al. (2021) reported that each 1 °F increase in average temperature was associated with a 4.0 % rise in traffic accident frequency in California and a 3.6 % rise in Arizona. One study reported that both cold and hot temperatures were associated with increased risks of motor vehicle crashes in New York, whereas only low temperatures showed an association in Chicago (Hou et al., 2022). In New York, each 1 °C decrease in mean daily temperature below −4.8 °C was associated with an 11.6 % increase in the relative risk of motor vehicle crashes, whereas each 1 °C increase above 26.1 °C was linked to a 1.6 % increase in risk. Lee et al. found that when the daily average temperature fell below −5.7 °C, each 1 °C decrease was associated with a 2.1 % increase in the incidence of traffic accident injuries in Seoul, Korea (Lee et al., 2014). A study conducted in Adelaide, Australia, found that both high ambient temperatures and elevated daily minimum temperatures were associated with an increased risk of road crashes, with moderately high temperatures contributing more to crash incidence than extreme heat (Li et al., 2023). In the subgroup study, people aged 18–45 years were identified as the most vulnerable group in heat environments, aligning with previous findings (Gu et al., 2025; Nazif-Munoz et al., 2025). Shanghai's status as a major employment hub is likely linked to this risk, as it attracts a large workforce of young and middle-aged individuals who may experience higher levels of heat exposure. Furthermore, evidence from Nazif-Munoz et al. (2025) suggests that heat stress may contribute to reductions in drivers' physiological functions, including cognitive performance and reaction times, which are potentially linked to higher accident risks during extreme heat conditions.

Our findings further revealed an association between cold environment exposure and elevated fall injury risks, which is more pronounced among women aged ≥46 years. In Australia, fall-related hip fracture hospitalization rates were higher in both men and women aged ≥75 years during periods of low ambient temperature (Turner et al., 2011). Several factors may contribute to this association. First, older adults experience reduced autonomic thermoregulatory capacity due to age-related physiological changes (Meade et al., 2020). Specifically, they exhibit lower metabolic heat production, which impairs their ability to effectively cope with cold stress (Kenney and Munce, 2003). Second, low temperatures frequently lead to icy or slippery outdoor surfaces. In the Shenkursk District (Unguryanu et al., 2020), outdoor fall injuries increased by 41 % and 63 % on days with loose dry snow or compact/wet snow cover, respectively, compared to snow-free days. Third, the higher prevalence of osteoporosis in females (Chandran et al., 2023) may further contribute to their increased vulnerability to fall injuries.

Furthermore, our study observed a positive association between extreme heat environment and assault risks, particularly among men aged 18–45 years. This finding is consistent with research from the United States, which reported that violent crime rates were 1.03 times higher at high temperatures compared to moderate temperatures (90th vs. 50th percentiles) (RR = 1.03; 95 % CI: 1.02, 1.04) and 1.04 times higher at moderate compared to cold temperatures (50th vs. 10th percentiles) (RR = 1.04; 95 % CI: 1.03, 1.06) (Heo et al., 2025). Similarly, a study in Korea observed a consistent rise in violent crimes as temperatures increased, with the highest relative risk observed at the 70th percentile of daily mean temperature (28.0 °C), where the RR was 1.11 (95 % CI: 1.09, 1.15) (Heo et al., 2024). Global burden data from 1990 to 2019 further highlight the heightened vulnerability of young men aged 15–29 years to self-harm and interpersonal violence under high-temperature conditions, with this risk remaining consistently high over time (Zhao et al., 2024). Many studies have found that elevated ambient temperatures can disrupt the homeostasis of neurotransmitters, particularly serotonin and dopamine, which may contribute to negative affect and increased impulsive aggression (Nakagawa and Ishiwata, 2021; Krakowski, 2003). Additionally, exposure to high temperatures has been shown to impair sleep quality (Zheng et al., 2019) and increase the likelihood of alcohol consumption (Liu et al., 2020), both of which are associated with a heightened risk of violent behavior.

Our study has several strengths. First, it is the first comprehensive investigation to examine the association between extreme temperatures and injury-related emergency ambulance dispatches in Shanghai, addressing a critical gap in regional epidemiological research. Second, stratified analyses by gender, age group, and injury mechanism enabled the identification of vulnerable subpopulations. These findings support the development of targeted public health interventions, such as early warning systems and infrastructure improvements, aimed at reducing risk among specific demographic groups. Third, in contrast to studies relying on hospital admission or outpatient records, the use of real-time ambulance dispatch data enables a more temporally precise assessment of temperature-related injury risks, thereby improving the validity of acute exposure-response analyses.

However, our study also has several limitations. First, as the study was conducted exclusively in Shanghai, the generalizability of the findings to other geographical regions may be constrained. Second, the initial diagnosis was formulated by the dispatching physician prior to the patient's hospital arrival, which could potentially introduce discrepancies when compared to the final clinical diagnosis. Third, this study only used data from fixed monitoring sites, but the temperature and pollutant exposures experienced by individuals may differ, which could be further investigated in future studies.

## 5 Conclusion

This study revealed differential associations between extreme temperatures and injury mechanisms. Extreme heat was associated with increased risks of traffic accidents and assault injuries, whereas extreme cold was linked to an increased risk of fall injuries. High temperatures were predominantly associated with elevated injury risks among men and young adults, whereas low temperatures showed a primary association with increased injury risk among the elderly. These findings may help identify vulnerable populations and guide prehospital emergency departments to develop targeted strategies for mitigating public health risks during extreme temperature events.

## CRediT authorship contribution statement

**Yan Hu:** Writing – original draft, Writing – review & editing, Methodology, Formal analysis, Data curation. **Li Peng:** Resources, Writing – review & editing, Conceptualization, Data curation. **Zhifeng Zhang:** Resources, Writing – review & editing, Conceptualization, Data curation. **Wenjie Lu:** Data curation, Formal analysis. **Haotian Jiang:** Conceptualization, Formal analysis. **Jiayue Zhu:** Methodology, Formal analysis. **Xu Liu:** Writing – review & editing, Conceptualization, Supervision, Funding acquisition.

## Funding

This study is supported by the Shanghai Municipal Health Commission, program “Three-year action plan for strengthening the construction of the public health system in Shanghai” (Grant/Award Number: GWVI-11.2-XD39).

## Declaration of competing interest

The authors declare that they have no known competing financial interests or personal relationships that could have appeared to influence the work reported in this paper.

## Data Availability

The authors do not have permission to share data.
