# Supplementary material for: Association between ambient temperature and injuries: A time series analysis using emergency ambulance dispatches in Shanghai
Source: Prev Med Rep. 2025 Jul 18;57:103177. doi: 10.1016/j.pmedr.2025.103177 (PMC12309581; doi:10.1016/j.pmedr.2025.103177)
Supplement: Supplementary material [file mmc1.docx]

**Supplementary Material**

**Table S1.** The associations between injury-related emergency ambulance dispatches and extreme temperatures over a lag period of seven days adjusted by confounders with different degree of freedom in Shanghai, 2016-2021.

| Confounders | Degree of freedom | Extreme low temperature  RR (95% CI) | Extreme high temperature  RR (95% CI) |
| --- | --- | --- | --- |
| Time | 7 | 1.02 (0.96, 1.09) | 0.99 (0.90, 1.08) |
|  | 8 | 1.05 (0.98, 1.12) | 1.08 (0.98, 1.19) |
|  | 9 | 1.06 (0.99, 1.14) | 1.05 (0.95, 1.16) |
| Wind speed | 3 | 1.05 (0.98, 1.12) | 1.08 (0.98, 1.19) |
|  | 4 | 1.05 (0.98, 1.12) | 1.08 (0.98, 1.19) |
|  | 5 | 1.05 (0.98, 1.12) | 1.08 (0.98, 1.19) |
| Relative humidity | 3 | 1.05 (0.98, 1.12) | 1.08 (0.98, 1.19) |
|  | 4 | 1.05 (0.98, 1.12) | 1.08 (0.98, 1.19) |
|  | 5 | 1.05 (0.98, 1.12) | 1.08 (0.98, 1.19) |
| PM_10_ | 3 | 1.05 (0.98, 1.12) | 1.08 (0.98, 1.19) |
|  | 4 | 1.05 (0.98, 1.12) | 1.09 (0.99, 1.20) |
|  | 5 | 1.05 (0.98, 1.12) | 1.09 (0.99, 1.20) |
| SO_2_ | 3 | 1.05 (0.98, 1.12) | 1.08 (0.98, 1.19) |
|  | 4 | 1.05 (0.98, 1.12) | 1.08 (0.98, 1.19) |
|  | 5 | 1.05 (0.98, 1.12) | 1.07 (0.98, 1.18) |
| CO | 3 | 1.05 (0.98, 1.12) | 1.08 (0.98, 1.19) |
|  | 4 | 1.05 (0.98, 1.12) | 1.08 (0.98, 1.19) |
|  | 5 | 1.05 (0.98, 1.12) | 1.08 (0.98, 1.19) |

Extreme low temperature was 1.2℃; Extreme high temperature was 32.6℃; RR: relative risk; CI: confidence intervals; PM_10_: Particulate matter with an aerodynamic diameter of less than 10 μm; SO_2_: Sulfur dioxide; CO: Carbon monoxide.

**Table S2.** Correlation coefficients between injury-related emergency ambulance dispatches and environment factors in Shanghai, 2016-2021.

|  | Injury-related emergency ambulance dispatches | Mean temperature | Relative humidity | Wind speed | PM_10_ | CO | SO_2_ |
| --- | --- | --- | --- | --- | --- | --- | --- |
| Injury-related emergency ambulance dispatches | 1 | 0.23^**^ | 0.06^**^ | -0.15^**^ | -0.16^**^ | -0.12^**^ | -0.43^**^ |
| Mean temperature |  | 1 | 0.24^**^ | -0.09^**^ | -0.29^**^ | -0.28^**^ | -0.26^**^ |
| Relative humidity |  |  | 1 | -0.17^**^ | -0.52^**^ | -0.04^*^ | -0.43^**^ |
| Wind speed |  |  |  | 1 | -0.08^**^ | -0.29^**^ | 0.05^*^ |
| PM_10_ |  |  |  |  | 1 | 0.60^**^ | 0.63^**^ |
| CO |  |  |  |  |  | 1 | 0.44^**^ |
| SO_2_ |  |  |  |  |  |  | 1 |

*$P<0.05$; ** $P<0.01$; PM_10_: Particulate matter with an aerodynamic diameter of less than 10 μm; CO: Carbon monoxide; SO_2_: Sulfur dioxide.


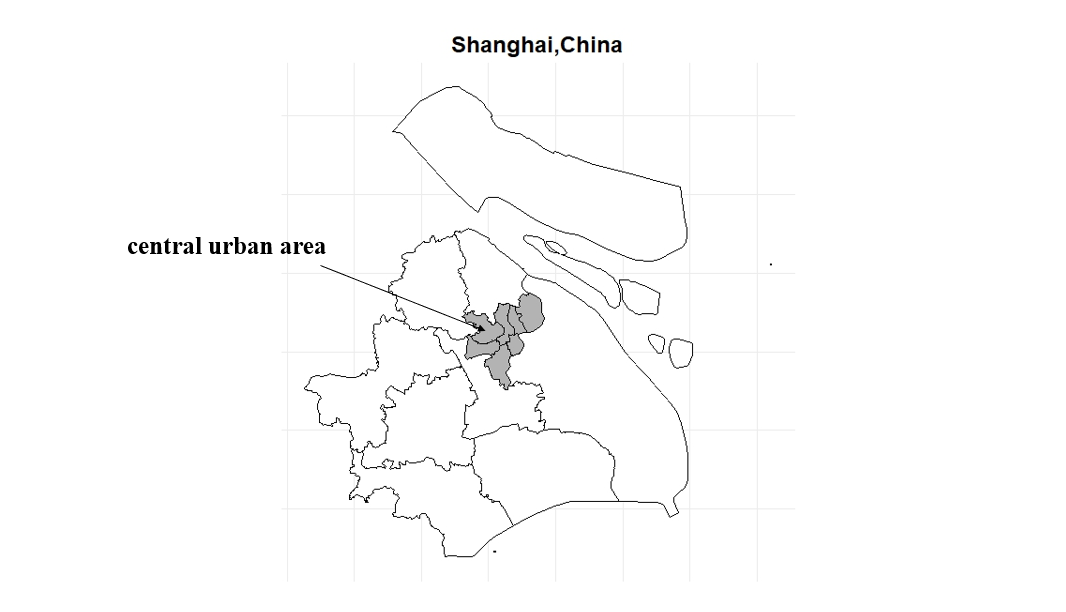


**Figure S1.** Locations of central urban area in Shanghai, China.
